# Current and emerging maintenance strategies after stem cell transplantation in children and adolescents with acute leukemias

**DOI:** 10.1016/j.omton.2026.201141

**Published:** 2026-01-29

**Authors:** Alexander W. Rankin, Amy K. Keating, Rolla F. Abu-Arja, Monica S. Thakar, Hemalatha G. Rangarajan

**Affiliations:** 1 Division of Hematology, Oncology, & Blood and Marrow Transplant, Nationwide Children’s Hospital, Columbus, OH 43205, USA; 2 Department of Pediatrics, The Ohio State University, Columbus, OH 43205, USA; 3 Department of Pediatric Oncology, Dana-Farber Cancer Institute, Division of Hematology/Oncology, Boston Children’s Hospital, Harvard Medical School, Boston, MA 02115, USA; 4 Clinical Research Division, Fred Hutchinson Cancer Center, Seattle, WA 98109, USA; 5 Department of Pediatrics, University of Washington School of Medicine, Seattle, WA 98109, USA

**Keywords:** MT: Special Issue - Advancements in pediatric cancer therapy, acute lymphoblastic leukemia, ALL, acute myeloid leukemia, AML, allogeneic stem cell transplantation, pediatrics, relapse prevention, maintenance

## Abstract

Hematopoietic stem cell transplantation (HSCT) can promote durable long-term remissions for children and adolescents with high-risk acute leukemias. While many patients with acute lymphoblastic leukemia (ALL) and acute myeloid leukemia (AML) can achieve a cure, post-HSCT relapse remains a possibility for many. Recent therapeutic advances, particularly in the realm of targeted therapeutics, have revolutionized both first-line and relapsed/refractory management strategies, opening the door to more personalized and potentially less toxic approaches to treatment. Many of these agents have also either been proposed or have been actively investigated as having a role in the post-HSCT setting. Post-HSCT relapse often carries a dismal prognosis, and early prophylactic intervention has in many cases been shown to improve outcomes. Herein, we comprehensively review maintenance strategies for prevention of post-HSCT relapse of ALL and AML, with a specific focus on pediatric and adolescent populations. While drawing on experience in adult patients, we highlight data specific to pediatrics where available and draw attention to areas where further research in children and adolescents is needed. Future efforts aimed at determining who will benefit from, when to initiate and discontinue, and what agent(s) to employ as maintenance will be crucial to optimizing post-HSCT outcomes.

## Graphical abstract

**Figure:**
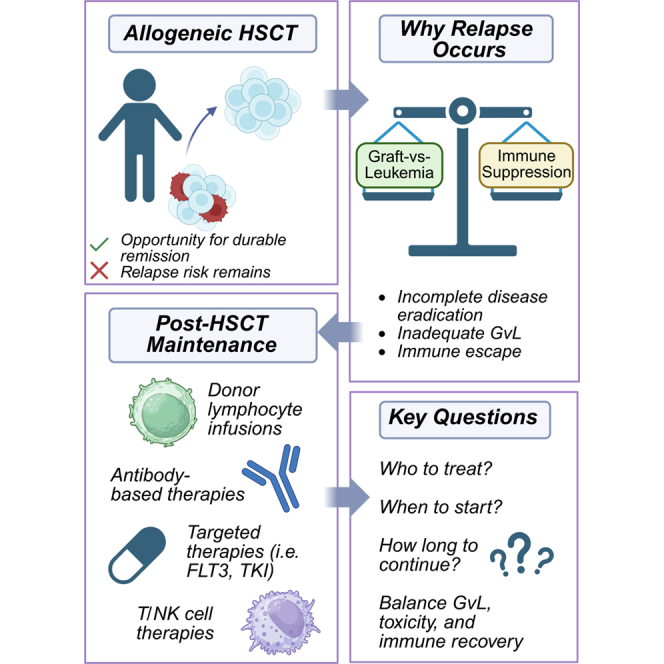

## Teaser

Strategies to prolong leukemia remission after allogeneic hematopoietic stem cell transplantation (HSCT) in children and adolescents are increasingly utilized. When employed post-HSCT, many targeted and immunotherapeutic approaches hold potential to improve outcomes both through direct antileukemic effects and synergistic immunomodulatory properties. Rankin and colleagues provide a comprehensive review of the available literature.

## Introduction

Acute leukemias represent the most common cancer diagnoses in children and adolescents. According to recent data, from 2018 to 2022 the 5-year incidence of leukemia in individuals <20 years of age was 4.8 per 100,000, with acute lymphoblastic leukemia (ALL) (3.8 per 100,000) being more common than acute myeloid leukemia (AML) (0.8 per 100,000).^1^ Significant treatment advances over the past several decades have led to improvements in survival outcomes, with patients with B cell ALL (B-ALL) collectively having a 5-year overall survival (OS) rate of around 90% or higher,^2^^,^^3^^,^^4^^,^^5^^,^^6^^,^^7^ whereas those with AML have 5-year OS rates of 50%–70% or lower for higher risk patients.^8^^,^^9^^,^^10^^,^^11^ Patients with T cell ALL (T-ALL), while historically having outcomes below patients with B-ALL, have shown comparable outcomes in the modern era.^12^^,^^13^ Despite what are often good long-term outcomes, prospects for durable remission in those with relapsed disease are much lower, with 5-year OS rates dipping to around 50%–60% for those with first relapse of B-ALL according to a Children’s Oncology Group (COG) study of cases from 1996 to 2014,^14^ and even lower for relapsed AML^15^ or T-ALL.^16^

Allogeneic hematopoietic stem cell transplantation (HSCT) can lead to long-term survival for patients with relapsed disease or higher risk upfront disease, such as those with high-risk cytogenetic features or poor response to chemotherapy. HSCT involves the replacement of the host hematopoietic system, which in the case of leukemia is impacted by disease, with stem cells and other immune cells from a healthy donor after ablation or near-ablation of the host bone marrow with chemotherapy with or without radiation. For certain diseases, autologously collected stem cells from the host can be used to rescue hematopoietic function after administration of high doses of chemotherapy. In the case of leukemia, however, allogeneic stem cells collected from a related or unrelated donor are preferred due to both the possible contamination of an autologously collected graft with malignant cells and the beneficial effect of alloreactive surveillance mediated by donor immune cells commonly termed the graft-versus-leukemia (GvL) effect.^17^

While cure can be achieved, post-HSCT relapse rates remain high, and survival rates are still not optimal. A recent study reported 5-year leukemia-free survival (LFS) after HSCT ranging from 33%-68% for ALL and 25%–78% for AML, with 5-year relapse incidence of 16%–47% and 14%–58%, respectively.^18^ Therefore, strategies for remission maintenance after HSCT are greatly needed, particularly in higher risk populations.

**Figure 1:**
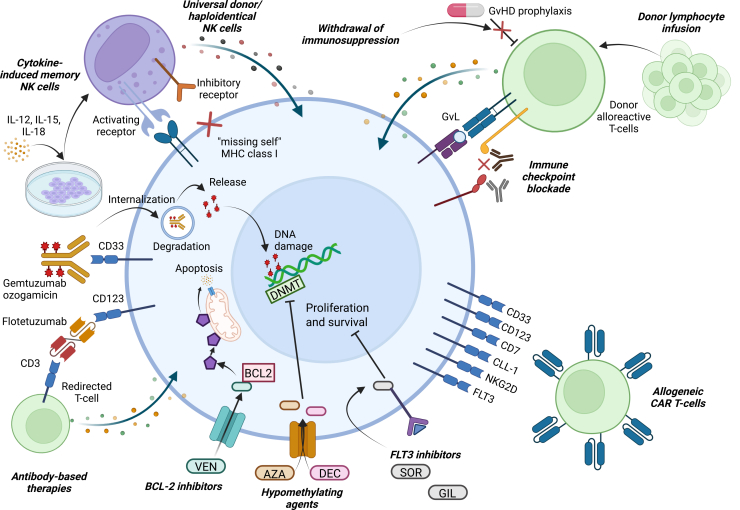
Post-HSCT therapeutic interventions for AML Overview of the various current and potential strategies for post-HSCT maintenance for children and young adults with acute myeloid leukemia (AML). Large blue cell in the center represents an AML cell, with various categories of therapeutic interventions detailed circumferentially around the periphery. Abbreviations: AZA, azacitidine; BCL2, B cell lymphoma 2; DEC, decitabine; DNA, deoxyribonucleic acid; DNMT, DNA methyltransferase; FLT3, FMS-like tyrosine kinase 3; GIL, gilteritinib; GvHD, graft-versus-host disease; GvL, graft-versus-leukemia; IL, interleukin; MHC, major histocompatibility complex; NK, natural killer; SOR, sorafenib; VEN, venetoclax.

**Figure 2:**
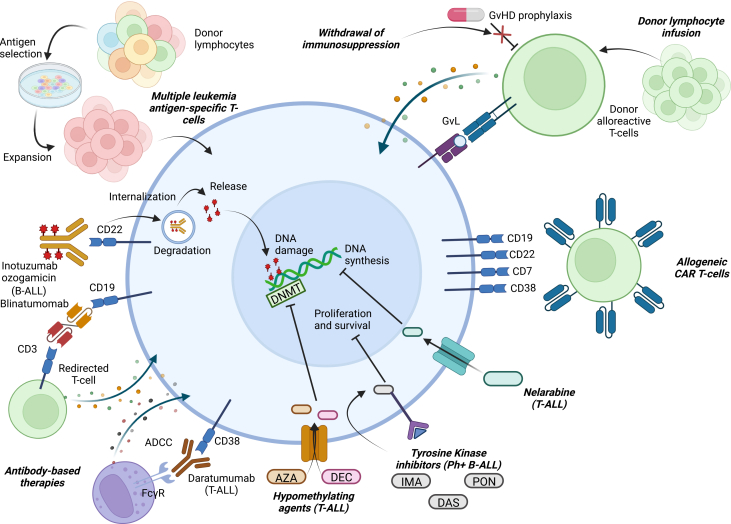
Post-HSCT therapeutic interventions for ALL Overview of the various current and potential strategies for post-HSCT maintenance for children and young adults with acute lymphoblastic leukemia (ALL). Large blue cell in the center represents an ALL cell, with various categories of therapeutic interventions detailed circumferentially around the periphery. Abbreviations: ADCC, antibody-dependent cellular cytotoxicity; AZA, azacitidine; DAS, dasatinib; DEC, decitabine; DNA, deoxyribonucleic acid; DNMT, DNA methyltransferase; FcγR, Fc gamma receptor; GvHD, graft-versus-host disease; GvL, graft-versus-leukemia; IMA, imatinib; PON, ponatinib.

To date, there is a lack of evidence-based strategies to promote durable remission in patients with acute leukemias after HSCT. This is particularly true in the pediatric and adolescent population, where there is a significant dearth of evidence. Herein, we will review the available evidence for post-HSCT remission maintenance strategies for AML (Figure 1) and ALL (Figure 2) in the pediatric and adolescent population and discuss some possible future directions. Due to the paucity of literature on post-HSCT maintenance in pediatric and adolescent patients, we will draw from evidence in adult patients where available, as well as preemptive approaches for the emergence of mixed chimerism or measurable residual disease (MRD), which often precede overt relapse.

As the rationale for therapy initiation post HSCT varies between studies, we will employ the terms “prophylactic” or “maintenance” to refer to an agent initiated before the emergence of MRD, mixed chimerism, or overt relapse, while the term “preemptive” will be used to describe an agent initiated after the emergence of MRD or mixed chimerism but prior to overt relapse. Additionally, use of the term MRD is somewhat vague and has evolved greatly over the past two decades. Multiple methods with varying sensitivities for detection of disease exist,^19^^,^^20^^,^^21^ and different studies may employ variable thresholds for defining MRD positivity. A standardized approach to measuring and defining the presence of MRD is needed, especially when evaluating the efficacy of interventions guided by its presence or absence, such as post-HSCT maintenance.

### Rationale for post-HSCT maintenance

While long-term durable remission after HSCT mediated by GvL is achievable in many patients with acute leukemias, the risk of relapse remains significant.^22^ The question as to which patients would benefit from maintenance therapy after HSCT remains debated. While some patients will relapse post-HSCT, others will achieve long-term remission, and whether the risk/benefit analysis would tilt toward all patients receiving some sort of maintenance post-HSCT or only those at highest risk of relapse remains unknown. Safety considerations regarding potential impacts on engraftment and immune reconstitution, along with how these effects may alter GvL, must be taken seriously. It is probable that maintenance approaches will provide the most benefit in higher risk populations, such as those with certain cytogenetic/molecular features,^23^ poor response to prior therapy,^24^ or pre-HSCT MRD.^25^ However, in the pediatric population, the calculus may favor more aggressive maintenance strategies given the general greater tolerability of therapy and longer remaining life expectancy as compared to older patients. This being said, care must be taken when extrapolating data from adults to children as toxicities may differ and data regarding late-onset side effects may not be available. The advent of targeted therapies will continue to expand the pool of patients who will have maintenance options available to them, but the populations that will benefit from each individual approach will be limited based on underlying disease characteristics, necessitating an increasingly personalized approach.

Certain biomarkers, such as the emergence of MRD^26^^,^^27^^,^^28^^,^^29^^,^^30^^,^^31^^,^^32^^,^^33^ or mixed chimerism,^34^^,^^35^^,^^36^^,^^37^^,^^38^ often herald overt relapse and can be acted upon through preemptive interventions; however, it is likely that maintenance approaches employed prior to the emergence of MRD or mixed chimerism may be more effective at exerting disease control. Many of the preemptive or maintenance strategies that have been reported in the literature have previously demonstrated efficacy in the upfront or relapse settings, and their use post HSCT has been extrapolated from these data. Additionally, there are several therapeutics that have shown promise in their potential to synergize with the GvL effect of HSCT, as discussed further, which may support their application in the post-HSCT setting.

### AML

#### Withdrawal of immunosuppression

The GvL effect mediated through alloreactive donor T-cell recognition of minor histocompatibility antigens is crucial in maintaining AML remission after HSCT.^39^^,^^40^ Immune escape from GvL is thought to play a major role in AML relapse,^41^ and suppression of donor T-cell activity through prolonged use of immunomodulatory medications post-HSCT may augment this.^42^ Withdrawal of immunosuppression (WIS) is a frequent strategy for emergence of MRD and/or mixed chimerism, and there is some evidence that early tapering may improve outcomes,^43^^,^^44^ particularly with modern graft-versus-host disease (GvHD) prevention strategies. While preemptive WIS in isolation may be of limited efficacy, its use in combination with other approaches may provide benefit. Regardless, an important consideration is that early WIS carries the risk of eliciting GvHD, in which case the need for augmented immunosuppression may erase any benefits derived from early WIS. Though often employed in higher risk pediatric patients with AML, debate remains regarding the particular scenarios in which early prophylactic WIS is of benefit.

#### Donor lymphocyte infusions

Boosting the GvL effect through donor lymphocyte infusion (DLI) has been attempted both in the treatment of overt AML relapse post-HSCT and in the preemptive management of impending relapse. While DLI has been an effective strategy for treating relapsed chronic myeloid leukemia, experience in AML has been more muted.^45^^,^^46^ However, if used as a preemptive approach for the emergence of MRD or mixed chimerism as opposed to overt relapse, benefit may be more pronounced.^47^^,^^48^ For example, one study in pediatric patients with AML showed that those who developed mixed chimerism post-HSCT and received either WIS or DLI had significantly improved event-free survival (EFS).^49^ A similar study reported that 6 of 13 patients who developed mixed chimerism and were treated with either WIS or DLI reestablished complete chimerism and maintained long-term remission.^50^ However, all of those who did not receive an intervention relapsed. These studies suggest that, in certain cases, early immunologic intervention prior to overt relapse can be an effective preemptive strategy to maintain ongoing remission.

An alternative to using DLI as preemptive management or treatment of relapse would be to employ it prophylactically for maintenance of remission. Here, while extrapolated from data in adult patients with less robust data in children, efficacy has been demonstrated with certain caveats. Several studies have reported this to be a feasible approach and demonstrated the ability to improve the durability of post-HSCT remissions,^47^^,^^51^^,^^52^^,^^53^^,^^54^ often in patients with high-risk disease. While there is no adequate data to determine the optimal dosing regimen for prophylactic infusion, most reports have involved a single dose. Importantly, any potential benefit must be weighed against the not insignificant risk of triggering GvHD, which may occur in as high as 50%–80% of patients. This risk has been mitigated to some degree by certain groups who have delayed infusion until day 120 in those who are off immunosuppression^53^^,^^55^^,^^56^; however, toxicity has been a limiting factor to more broad application of this approach. Additionally, donor type may influence outcomes, although data do not exist to clearly inform the decision to pursue DLI based on donor type alone. Some studies have suggested lower risk of GvHD when using matched related donors compared to unrelated donors.^53^^,^^56^^,^^57^ However, related donors may also conceivably generate less alloreactivity and GvL, leading to higher relapse risk.^52^^,^^53^^,^^56^ While donor impact on survival outcomes is not proven, different dosing regimens are often employed for related and unrelated donors to account for expected differences in toxicity.^50^^,^^52^

Additional strategies to improve the efficacy of DLI may involve combinatorial approaches utilizing agents with known immunomodulatory properties, such as hypomethylating agents^58^^,^^59^^,^^60^^,^^61^ (discussed further), or cellular manipulation strategies to promote efficacy while limiting GvHD, such as TCRαβ+ T cell depletion.^62^^,^^63^ Studies specific to pediatric patients are needed to determine if data from adult patients can appropriately be extrapolated to a younger population.

#### Chimeric antigen receptor T cells

While chimeric antigen receptor (CAR) T cells have demonstrated remarkable efficacy in relapsed/refractory B-ALL, the same degree of success has not been seen in AML. CAR T cells directed at multiple targets are currently under preclinical or clinical investigation, including CD33,^64^^,^^65^^,^^66^^,^^67^ CD123,^68^^,^^69^ CD7,^70^^,^^71^ CLL-1,^72^^,^^73^^,^^74^ NKG2D,^75^ and FLT3^76^^,^^77^; however, to date clinical efficacy is not proven. Allogeneic donor-derived CAR T cells are also under investigation for relapsed disease after HSCT.^78^^,^^79^ Future studies may consider donor-derived CAR T cells as a post-HSCT maintenance option.

#### Natural killer cells

Natural killer (NK) cells, which play a key role in promoting GvL without significantly driving GvHD, represent a promising cellular therapy approach to post-HSCT maintenance. Several studies have demonstrated the feasibility of using NK cells in the treatment of relapsed disease with mixed outcomes.^80^^,^^81^ The ongoing KARMA trial (NCT05503134) is a single-center phase 1/2 study evaluating universal donor NK cells combined with chemotherapy for the treatment of relapsed/refractory AML in young adult patients aged 18–25. Outside of the HSCT setting, Nguyen et al. evaluated the use of prophylactic haploidentical killer cell immunoglobulin-like receptor (KIR)-mismatched NK cells for consolidation of remission in pediatric patients.^82^ Twenty-one patients in first complete remission (CR) with a median age of 6.0 years received NK cell infusions following conditioning with no reported major toxicities, including GvHD. The majority displayed transient NK cell engraftment, but NK cells were not found to decrease the incidence of relapse or improve EFS or OS over chemotherapy alone. The ongoing EXCEL trial (NCT04836390) is a pilot phase 2 study evaluating the use of prophylactic *ex*-*vivo*-expanded haploidentical NK cells as consolidation of remission in the post-HSCT setting in pediatric and young adult patients. No results have been reported to date.

One limitation of NK cell therapy has been a lack of *in vivo* persistence. Cytokine-induced memory-like (CIML)-NK cells, generated through *ex vivo* culture with cytokines such as interleukin (IL)-12, IL-15, and IL-18, may further improve upon NK cell antileukemia efficacy and enhance persistence.^83^ There are several ongoing clinical trials utilizing this approach in pediatric patients, including for relapsed/refractory disease, post-HSCT relapse, and post-HSCT remission maintenance. Bednarski et al. have reported on the treatment of 9 pediatric/young adult patients with post-HSCT relapse using donor-derived CIML-NK cells in combination with DLI (NCT03068819).^84^ NK cells expanded and displayed persistent functional response without an increased incidence of GvHD, and 4/8 evaluable patients achieved a CR. Two of those patients maintained a durable remission for >3 months. Further data from ongoing efforts are anticipated to help inform the efficacy of post-HSCT NK cell approaches in the pediatric and adolescent population.

#### Other cellular therapies

Other modes of cellular therapy that may hold potential in the post-HSCT setting include T cell receptor (TCR) T cell therapies^85^^,^^86^ or cytokine-induced killer (CIK) cells. One group demonstrated that EBV-specific donor CD8^+^ T cells transduced with WT1-specific TCRs can be beneficial in maintaining GvL without inducing GvHD when employed prophylactically.^87^ Another group infused leukemia antigen-specific T cells (PRAME, WT1, survivin, and NY-ESO-1) prophylactically with a tolerable safety profile and encouraging efficacy.^88^ CD8^+^ T cells targeting the minor histocompatibility antigen HA-1 have also been proposed in the post-HSCT setting.^89^^,^^90^^,^^91^ Regarding CIK cells, a recent study reported on prophylactic infusion post HSCT for myeloid neoplasms but noted similar relapse and survival rates compared to historical controls.^92^

#### Antibody-based therapies

The CD33-targeted antibody-drug conjugate gemtuzumab ozogamicin (GO) has altered the landscape of pediatric AML treatment in recent years. Various studies have now been published regarding its utility in treating newly diagnosed AML^93^^,^^94^^,^^95^^,^^96^ as well as relapsed/refractory disease.^97^ Its use in the post-HSCT setting as both salvage combined with chemotherapy^98^ and prophylactically^99^^,^^100^^,^^101^ have been explored with limited data. Potential concerns with using GO in the post-HSCT setting include its association with sinusoidal obstructive syndrome (SOS) as well as concerns about toxicity to engrafting hematopoietic stem cells. As a novel approach to the latter problem, an ongoing clinical trial is investigating the use of GO prophylactically as post-HSCT maintenance following therapy with VOR33, an allogeneic CRISPR-Cas9-edited HSCT product lacking the CD33 protein (NCT04849910).^102^

Flotetuzumab is a dual-affinity re-targeting antibody that binds CD123 on AML cells and CD3^+^ T cells, working to redirect T cell cytotoxicity against AML. It has shown encouraging activity in adults with relapsed/refractory AML,^103^^,^^104^ and the COG PEPN1812 phase 1 study demonstrated safety and tolerability in children with an overall response rate of 20% at the recommended phase 2 dose.^105^ There is no data on its use in the post-HSCT setting, although a phase 1 trial for post-HSCT relapse (NCT05506956) has recently been completed. Pivekimab sunirine (IMGN632)^106^ and tagraxofusp^107^ are two additional CD123-targeting agents being investigated in the early-phase setting for AML that may hold future potential for post-HSCT application.

AML patients with the CBF2AT3-GLIS2 oncogenic fusion (“RAM” phenotype) have a particularly poor prognosis. STRO-002, an antibody-drug conjugate targeting FR-α expressed on RAM phenotype AML, has shown promising activity in relapsed/refractory disease and seems to be more effective in those with low tumor burden,^108^ again suggesting a potential application post-HSCT as preemptive or prophylactic therapy. Indeed, while published data are not yet available, given the extraordinarily high risk of relapse, many centers have unpublished experience safely utilizing STRO-002 prophylactically as post-HSCT maintenance therapy. Investigation of ELU001,^109^ a similar molecule targeting CBF2AT3-GLIS2 AML was also planned, but the trial was withdrawn prior to enrollment (NCT05622591).

#### Hypomethylating agents

Agents such as azacitidine and decitabine have been studied extensively in the post-HSCT setting in adults as treatment of relapse^110^^,^^111^^,^^112^ as well as preemptive^113^ and maintenance^51^^,^^114^^,^^115^^,^^116^^,^^117^ therapies. Several studies have demonstrated an immunomodulatory role for hypomethylating agents in terms of increasing the expression of tumor antigens such as WT1^118^ and MAGE,^119^^,^^120^ promoting CD8^+^ cytotoxic T cell responses, and augmenting the expansion of regulatory T cells.^121^^,^^122^^,^^123^ In adult patients, most studies to date have demonstrated benefit in terms of improving survival and decreasing relapse rate when azacitidine or decitabine are used as prophylaxis post-HSCT,^116^^,^^117^^,^^124^^,^^125^^,^^126^ but some have failed to show improvement in outcomes.^127^^,^^128^^,^^129^

In the pediatric population, studies have begun to investigate the feasibility and efficacy of using hypomethylating agents in combination with other agents in relapsed patients.^130^^,^^131^^,^^132^^,^^133^ There are also several studies that have explored the use of azacitidine or decitabine prophylactically as post-HSCT maintenance in pediatric patients. Gao et al. conducted a phase 2 randomized controlled trial using the combination of prophylactic decitabine and granulocyte colony-stimulating factor (G-CSF) in patients with AML who were MRD negative after HSCT.^134^ While the study enrolled mostly adult patients, approximately 25% were <20 years of age. They found a 2-year cumulative incidence of relapse of 15% in the study arm vs. 38.3% in the control arm with incidence of chronic GvHD being 34.0% and 48.2%, respectively. They also noted increased proportions of NK cells, CD8^+^ T cells, and regulatory T cells in the study arm.

Tamura et al. presented a case series of 3 high-risk pediatric AML patients who were in remission post HSCT and received low-dose azacitidine prophylactically.^135^ The approach was feasible, and all remained in remission 13–41 months after HSCT with one developing acute GvHD. Recently, Booth et al. published their single-center experience using prophylactic low-dose azacitidine in combination with DLI as post-HSCT maintenance.^59^ Compared to historical controls, patients who received maintenance therapy had a 2-year LFS of 88.2% vs. 61.5% (*p* = 0.06), OS of 88.2% vs. 69.2% (*p* = 0.15), rate of grade II–IV acute GvHD of 38% vs. 30% (*p* = 0.51), and rate of moderate-severe chronic GvHD of 13% vs. 17% (*p* = 0.68). Given the experience in adult patients, application to pediatric patients is reasonable; however, larger prospective studies are needed.

#### B cell lymphoma 2 inhibitors

Venetoclax, a B cell lymphoma 2 (BCL-2) inhbitor, has been used effectively, particularly in combination with azacitidine, to target leukemia stem cells in adult patients with AML.^136^^,^^137^ There are also data to suggest that venetoclax is safe and tolerable as post-HSCT maintenance in adults.^138^^,^^139^ In pediatrics, the data are more limited, but available evidence is encouraging.^140^^,^^141^^,^^142^ Although no studies have been published on its use prophylactically as post-HSCT maintenance in pediatrics, the VIALE-T trial (NCT04161885) will evaluate the combination of venetoclax and azacitidine as maintenance in patients down to the age of 12 years.

#### FMS-like tyrosine kinase 3 inhibitors

Data suggest that in patients with FMS-like tyrosine kinase 3 (FLT3)-mutant AML, either with internal tandem duplication (ITD) or mutation in the tyrosine kinase domain (TKD), the addition of a targeted inhibitor is safe and improves outcomes in both adults and pediatrics.^10^^,^^143^^,^^144^ Indeed, in the post-HSCT setting, the ability of FLT3 inhibitors to induce alloimmune effects that may enhance GvL,^145^^,^^146^^,^^147^ which may be even more pronounced in combination with other immunotherapeutic approaches such as DLI,^148^ provides a strong rationale for their use.

Multiple studies in adult patients have suggested the potential of prophylactic post-HSCT maintenance with FLT3 inhibitors^149^^,^^150^^,^^151^^,^^152^^,^^153^^,^^154^ to improve remission duration. A recent randomized study showed that preemptive post-HSCT gilteritinib was beneficial in FLT3-ITD patients with either pre- or post-HSCT detectable MRD; however, no benefit was seen in those without detectable MRD.^155^ In pediatrics, the experience is less robust. Battipaglia et al. studied sorafenib prophylaxis in patients down to the age of 15 years, but the median age of subjects was 46 years.^156^ A retrospective study by Tarlock et al. analyzed the use of post-HSCT sorafenib in 15 pediatric patients, 6 of whom were treated as prophylaxis.^157^ All patients who received sorafenib preemptively either for pre-HSCT MRD or emergence of post-HSCT MRD were alive and in CR at 48 months. Pollard et al. also retrospectively examined the use of sorafenib post-HSCT in 13 patients, 8 of whom were treated prophylactically.^158^

Use in pediatrics as maintenance is often pursued and is reasonable given adult data and growing experience in children. It also remains unknown which FLT3 inhibitor, if any, is superior for use as maintenance and the optimal duration of maintenance is yet to be elucidated, although the European Society for Bone Marrow Transplantation (EBMT) recommendation is to continue for 2 years post-HSCT.^159^

#### Other targeted therapies

Several other targeted agents are being actively investigated or may have future potential as maintenance in the post-HSCT setting. Histone deacetylase inhibitors, such as hypomethylating agents and FLT3 inhibitors, may have some level of immunomodulatory effect via control of cytokine secretion and transcriptional modification of genes regulating immune checkpoint molecules.^160^ Post-HSCT, panobinostat maintenance has resulted in survival rates that compare favorably to historical data.^161^ A separate phase 3 trial in adult patients has completed accrual (NCT04326764), and a phase 1 trial of vorinostat in combination with azacitidine is currently recruiting (NCT03843528).

Isocitrate dehydrogenase (IDH) mutations can lead to epigenetic dysregulation and impairment of cellular differentiation in AML.^162^ IDH inhibitors have shown some efficacy for relapsed/refractory AML in adults,^163^ and both enasidenib^164^ and ivosidenib^165^ have been studied prophylactically as post-HSCT maintenance with some preliminary signals of efficacy. However, IDH1/2 mutations are rare in pediatric AML,^166^ making it doubtful they would have a large impact in younger populations.

The menin inhibitor revumenib received Food and Drug Administration (FDA) approval in 2024 for relapsed/refractory KMT2a-rearranged AML in children and adults,^167^^,^^168^ and Zucenka et al. reported on its prophylactic or preemptive use post-HSCT for patients enrolled in the pivotal AUGMENT-1 trial.^169^ Nine patients enrolled in the trial went on to receive revumenib after HSCT if in morphological CR, resuming between 59 and 180 days after. CR was maintained in 6 patients, with one patient who was MRD positive becoming MRD negative after initiating therapy. Its use post-HSCT warrants further study.^170^

Hedgehog pathway signaling is important for maintenance of leukemia stem cell populations, and pathway inhibition may mitigate leukemia development and sensitize leukemia stem cells to chemotherapy.^171^ However, the Hedgehog pathway inhibitor glasdegib to date has not shown efficacy as post-HSCT maintenance.^172^

CD47, an anti-phagocytic transmembrane protein, is highly expressed on AML leukemia stem cells, and increased expression is associated with worse prognosis.^173^ Magrolimab is an anti-CD47 monoclonal antibody that has not yet been studied in the post-HSCT setting but has shown some promise as part of combination therapy in early trials for *de novo* AML.^174^ However, results from more recent trials have been more muted.^175^^,^^176^

#### Checkpoint blockade

Targeting inhibitory checkpoint molecules, specifically programmed cell death protein (PD)-1/PD-ligand (L)1 and cytotoxic T lymphocyte-associated protein (CTLA)-4, may augment the antileukemia GvL effect after HSCT. Davids et al. showed that the administration of ipilimumab for patients with recurrent hematologic malignancies after HSCT led to some durable responses, including 2 patients with extramedullary AML.^177^ However, immune-mediated adverse events including acute GvHD occurred, and this has also been demonstrated with the use of nivolumab^178^^,^^179^^,^^180^ and pembrolizumab^181^ with only moderate antitumor activity. Whether checkpoint inhibition as maintenance in patients without significant tumor burden would be more efficacious and tolerable is unknown, and combination therapy with other agents to enhance GvL without promoting GvHD may provide theoretical benefit. Several trials are continuing to investigate the use of checkpoint inhibition for AML post-HSCT (NCT03286114, NCT03600155, and NCT02846376).

### ALL

#### Withdrawal of immunosuppression

While the GvL effect is generally thought to be more potent in AML than ALL, WIS to enhance graft activity against the leukemia has been employed in ALL as well. Often used as a preemptive strategy for the management of mixed chimerism, it is employed alone or in combination with DLI to enhance GvL. Several studies in pediatrics have suggested that this can be an efficacious approach at restoring full donor chimerism and decreasing relapse.^37^^,^^182^^,^^183^ While there is little in the literature studying prophylactic early WIS in patients with ALL, relapse risk often informs clinicians’ decision to begin immunosuppression taper early in these patients, with higher risk patients often beginning withdrawal earlier.^184^ Ultimately, however, data are limited and not sufficient to determine the true efficacy of early WIS prior to the emergence of mixed chimerism or overt relapse.

#### Donor lymphocyte infusions

The utility of DLI has been limited in ALL. Multiple early studies in adults suggested that response rates were low in patients with relapse,^45^^,^^185^ and poor efficacy was linked in one study to the limited proliferative capacity of ALL-specific alloreactive T cells and was dependent on the HLA disparity between the donor and the patient.^186^ Additional early concerns had surrounded the high rates of GvHD associated with DLI; however, a recent study using a relatively large cohort of pediatric and young adult patients suggested rates comparable to the general HSCT population (acute: 19.4%, chronic: 9.2%).^187^ Further, while some studies have begun to investigate the utility of prophylactic DLI for post-HSCT ALL relapse prevention and have shown some benefit, many studies include both patients with AML and ALL, making it difficult to parse differential outcomes between the two different disease processes.^188^^,^^189^^,^^190^

In pediatrics, the outcomes using DLI for post-HSCT ALL relapse have been similarly underwhelming. Levine et al. reported outcomes of 49 children who received DLI for relapse after HSCT, including 18 with ALL.^191^ Prognosis was poor overall, especially in those who relapsed early (<6 months after HSCT) and in those with ALL, among whom 13/18 died of leukemia without achieving remission. Additionally, of the 4 who achieved remission, 2 later died from relapse and 1 from chronic GvHD. When all patients were compared to a matched cohort that did not receive DLI, no difference was found in survival between those who received DLI and those who did not. In contrast, a single-center retrospective study reported a 71% 5-year OS for children with ALL who received DLI post-HSCT, higher than the 32% for patients with AML.^192^ However, only 7 patients with ALL were included in this study.

In the preemptive setting, a retrospective study from Germany reported that there was no difference in relapse rates or survival when comparing pediatric patients with ALL who received DLI for mixed chimerism or emergence of MRD and those who did not.^193^ Gruhn et al. recently presented results of 34 pediatric patients (10 of whom had ALL), the majority of whom received preemptive or prophylactic DLI.^194^ While they did not separate response by underlying disease, 83% of those treated preemptively did not develop overt relapse, and none of the patients treated prophylactically relapsed. Interestingly, DLI was combined in several patients with blinatumomab, inotuzumab, and zoledronic acid to promote TCRγδ+ T cell cytotoxicity, all of which could be interesting avenues for future combinatorial studies.

In short, while efficacy for preemptive or salvage therapy appears to be minimal, the appropriate role for post-HSCT DLI as relapse prophylaxis remains to be elucidated.

#### Tyrosine kinase inhibitors

Maintenance with tyrosine kinase inhibitors (TKIs) is frequently pursued after HSCT for patients with Philadelphia chromosome-positive (Ph+) B-ALL, and many would now consider it to be standard of care. A study by the EBMT Acute Leukemia Working Party showed that the use of TKIs prophylactically after HSCT was associated with improved LFS and OS as well as a lower incidence of relapse.^195^ Interestingly, in multivariate analysis post-HSCT, TKI use was associated with a lower incidence of GvHD. First-,^196^ second-,^197^^,^^198^ and third-^199^^,^^200^ generation TKIs have all shown tolerability and efficacy in the post-HSCT setting; however, evidence on the best choice of TKI is mixed.^201^ A retrospective comparison of imatinib and dasatinib as post-HSCT prophylaxis did not show difference in outcomes but less tolerability of dasatinib.^202^ However, a randomized trial in children found dasatinib to be superior to imatinib when added to upfront chemotherapy.^203^ The EBMT has produced consensus guidelines on TKI use post-HSCT, recommending imatinib for most patients and dasatinib in cases of imatinib resistance, early molecular relapse, or significantly elevated BCR-ABL transcripts (>10^4^).^204^ Duration of therapy is generally at least 24 months, as this has been shown to reduce relapse risk.^205^

In pediatrics specifically, the COG AALL0031 and the intergroup EsPhALL2010 studies both demonstrated the utility of imatinib as part of upfront therapy for patients with Ph+ ALL, with the addition of a TKI to intensive chemotherapy resulting in outcomes similar to consolidative HSCT.^206^^,^^207^ Additionally, CA180-372/COG AALL1122 showed that dasatinib plus EsPhALL chemotherapy is effective for pediatric Ph+ ALL,^208^ and a retrospective study from the Japan Children’s Cancer Group suggested that ponatinib was safe and effective as well.^209^ However, when it comes to post-HSCT maintenance, there is a lack of pediatric-specific data, and usage is largely extrapolated from data in adults.

The use of sensitive molecular testing such as low-density microarray and reverse-transcription polymerase chain reaction (RT-PCR) has led to the increased recognition of B-ALL subtypes harboring kinase-activating mutations that result in a gene expression profile similar to Ph+ ALL but without the canonical BCR-ABL fusion protein.^210^ This “Ph-like” ALL may also be targetable with TKIs (as with ABL-class fusions) or other molecularly targeted therapies such as JAK inhibition. While these agents are increasingly used in the upfront or relapsed setting, only isolated case reports have been published on their use post HSCT,^211^^,^^212^ although unpublished experience is growing across a number of centers.

#### Blinatumomab

Blinatumomab is a bispecific T cell engager that targets CD19 and CD3 and works to engage and activate cytotoxic T cells and bridge them with CD19^+^ B cells to promote tumor killing. The efficacy of blinatumomab for both relapsed^213^^,^^214^ B-ALL and upfront management^215^^,^^216^ is well documented, and its inclusion in upfront chemotherapy regimens is becoming standard. In pediatrics, a recent study also suggested that post-HSCT outcomes are improved when blinatumomab is used as pre-HSCT remission consolidation as opposed to chemotherapy.^217^ In the post-HSCT relapse setting, there is evidence that blinatumomab can lead to meaningful responses through restoration of GvL in adult patients without apparent increase in GvHD risk.^218^^,^^219^ Combination with DLI may hold potential to enhance this further; however, evidence to date is limited and inconclusive.^220^^,^^221^

Regarding its use as prophylaxis for MRD-negative remission post-HSCT, several recent studies have suggested tolerability and efficacy in high-risk patients across a variety of dosing regimens.^222^^,^^223^ Two of these studies additionally demonstrated that those who were T cell deficient or had lower peripheral blood CD8:CD4 ratios and upregulation of inhibitory checkpoint molecules were associated with poorer response,^224^^,^^225^ suggesting that strategies to address these may enhance efficacy. While rates of GvHD were acceptable in these studies, care would need to be taken if considering addition of therapies such as checkpoint inhibitors given the risk of promoting T cell alloreactivity.

Huang et al. recently retrospectively reviewed their experience using prophylactic blinatumomab as post-HSCT maintenance in 21 patients, 6 of whom were under the age of 20.^226^ Blinatumomab was tolerable, with grade 1–2 GvHD occurring in 28.6% and grade 3–4 occurring in 4.8%. Five patients developed chronic GvHD. Relapse rate was only 6.4%, and 1-year OS, EFS, and GvHD/relapse-free survival (GRFS) were 81.6%, 82.1%, and 82.5%. Review of the data reveals that of the 6 patients <20 years of age, 1 with pre-HSCT MRD positivity relapsed and died while the others appeared to have remained in remission. Sakaguchi et al. are conducting a multi-center phase 1/2 study evaluating the use of blinatumomab as post-HSCT maintenance in children and young adults,^227^ which should help to clarify its role in the pediatric population.

As CD19-targeted therapies become more incorporated into the upfront and relapsed settings, both with increasing usage of blinatumomab and CD19 CAR T cells, it remains to be seen how this would impact the efficacy of further CD19 targeting post-HSCT, and consideration of additional antigenic targets may be necessary.

#### Inotuzumab

Little data are available on the application of the CD22-targeting antibody-drug conjugate inotuzumab ozogamicin for post-HSCT prophylaxis, and the association of inotuzumab with SOS may hamper enthusiasm for its use. However, a recent phase 1 study reported a tolerable safety profile for inotuzumab as maintenance with no episodes of SOS in a dose-escalation model across 18 patients.^228^ While an advantage of inotuzumab over blinatumomab may be that the latter requires endogenous cytotoxic T cells for efficacy (which may be rare or poorly functional in a post-HSCT reconstituting immune system) while the former does not, more data are needed to inform the safety and efficacy of inotuzumab in this setting.

#### Cellular therapy

The efficacy of autologous CD19 CAR T cells for relapsed/refractory B-ALL, including post-HSCT relapse,^229^^,^^230^ lends credence to the idea that they could be employed post-HSCT as a relapse prevention strategy. Allogeneic manufacturing platforms open the window for donor-derived infusions, although GvHD needs to be considered with this approach.^231^^,^^232^^,^^233^ Lu et al. treated 23 patients with what was termed prophylactic donor-derived CAR T cell infusion after HSCT, although 7 patients had active MRD.^234^ Notably, the infusions were tolerable with no severe cytokine release syndrome (CRS) or immune effector cell-associated neurotoxicity syndrome (ICANS), and only 3 patients developed acute GvHD, although hematotoxicity was prevalent. The 2-year incidence of relapse was 5.6%, compared to 28.8% in a contemporary control cohort. While further work is needed, these results are encouraging.

A plethora of additional targets for relapse prophylaxis, both for B and T cell ALL, could be envisioned, including but not limited to CD22,^235^^,^^236^ CD7,^237^^,^^238^ and CD38.^239^ Additionally, donor-derived leukemia-specific T cells targeting multiple antigens (PRAME, WT1, and survivin) may be a promising maintenance strategy after HSCT,^240^ while other TCR-, TCRγδ+-, or NK-based therapies remain to be explored.

#### T cell ALL

While less common than B-ALL, T-ALL also carries a high risk of relapse post-HSCT, and thus effective maintenance strategies are an area of unmet need. Although data are less robust, likely related to smaller patient numbers, several strategies deserve mention.

As in AML, hypomethylating agents have been studied as post-HSCT maintenance for patients with ALL. Two Chinese studies in adult patients suggested that decitabine after HSCT may provide benefit in reducing incidence of relapse, particularly in patients with T-ALL.^241^^,^^242^ The larger of the two studies reported a 3-year incidence of relapse of 19.6% vs. 36.1% in those who received decitabine vs. controls and was even lower in those with T-ALL who received decitabine (11.7%). BCL-2 inhibition may be another avenue for relapse prophylaxis in T-ALL, as BCL-2 is highly expressed in early T cell precursors and T-ALL cell lines do display sensitivity to BCL-2 inhibition.^243^ Hassan et al. described the prophylactic treatment of 4 T-ALL patients with post-HSCT venetoclax/azacitidine and noted that all four maintained remission with a median follow-up time of 15 months.^244^

Data on other strategies specifically for T-ALL are lacking, however. Nelarabine, which has been shown to improve upfront survival in pediatric patients with T-ALL,^245^ has also shown efficacy as salvage in the post-HSCT setting^246^ but has not been reported as a maintenance strategy. Bortezomib has also demonstrated efficacy in the upfront and relapse setting,^247^^,^^248^ and CD38 targeting with daratumumab has been investigated for relapsed/refractory disease as well.^249^^,^^250^ Data on post-HSCT use are currently absent; however, an ongoing clinical trial (NCT04972942) is investigating the use of daratumumab as post-HSCT maintenance in children and young adults with high-risk T cell leukemia and lymphoma. While CAR T cells for T-ALL may be complicated by fratricide and a risk of long-standing T cell lymphopenia, innovative engineering approaches have opened the door for targeting T lineage antigens, such as CD7.^251^ Donor-derived allogeneic approaches for relapse prevention should be subjects of future research.

### Impact on post-HSCT immune reconstitution

Regardless of the post-HSCT intervention employed, careful consideration of the potential impacts on immune reconstitution is crucial. DLI can lead to the conversion of mixed donor chimerism to full chimerism and improve T cell reconstitution^252^^,^^253^ but may also drive increased alloreactivity and GvHD, especially at higher doses and when given earlier post HSCT,^254^ which may distort immune recovery. Prophylactic NK cell infusions have expectedly been associated with improved NK cell reconstitution^255^^,^^256^^,^^257^ and may specifically be advantageous in the context of post-transplant cyclophosphamide, which can ablate proliferating and alloreactive NK cell populations.^258^^,^^259^

Agents with myelosuppressive properties may conceivably lead to delays in immune reconstitution through suppression of hematopoiesis, and dosage holds and reductions are commonly needed to address hematotoxicity. However, to our knowledge, systematic evaluation of the impact of more commonly used agents such as hypomethylating agents, FLT3 inhibitors, and TKIs on immune recovery is not available. Similarly, how agents such as inotuzumab and blinatumomab impact immune reconstitution beyond inducing cytopenias is not well described, although in the case of blinatumomab, it has been shown that efficacy post HSCT may be constrained by the recovering T cell repertoire.^224^

Understanding of the impact of post-HSCT maintenance strategies on immune recovery would provide deeper mechanistic insights into effects on GvL and toxicity as well as shed light on potential concerns regarding post-HSCT infections and revaccination. As such, further study in this area is much needed.

## Conclusions and future directions

The curative potential of allogeneic HSCT for children and adolescents with acute leukemias continues to be limited by relapse. The use of post-HSCT maintenance has made strides in promoting durable remissions in patients with “druggable” targets, such as FLT3-mutated AML or Ph+ ALL. However, this still leaves most patients without evidence-based options for post-HSCT remission prophylaxis. A select list of active clinical trials is shown in Table 1. Approaches to enhance GvL, such as WIS ± DLI, have generated only modest efficacy at best and can be limited by the development of GvHD. Allogeneic cellular therapy approaches, such as CAR T cells or NK cells, are promising, but the current focus for these and many other therapies is on salvage potential for patients with active disease as opposed to relapse prevention. Additionally, even for interventions that have shown efficacy in adults, data in pediatrics specifically can be quite hard to come by, a problem often faced by pediatric hematologists/oncologists when providing evidence-based counseling to families. While extrapolating from data in adult patients may provide insight, it is not clear that this is always appropriate, and trials specifically targeted at the pediatric and adolescent population are needed.

**Table 1:** Select clinical trials for post-HSCT maintenance currently recruiting

ClinicalTrials.gov ID	Study name	Disease	Age	Sponsor
NCT06158828	Pilot Study of Memory-like Natural Killer (ML NK) Cells After TCRαβ T cell Depleted Haploidentical Transplant in AML (ABCD-NK)	AML	<30 years	Washington University School of Medicine
NCT05686538	Innate Donor Effector Allogeneic Lymphocyte Infusion After Stem Cell Transplantation: the IDEAL Trial (IDEAL)	AML, MDS	≥18 years	Rigshospitalet, Denmark
NCT06765915	Avapritinib Maintenance for AML With KIT Mutations	AML	≥14 years	Ruijin Hospital
NCT03286530	Ruxolitinib + Allogeneic Stem Cell Transplantation in AML	AML	60–80 years	Massachusetts General Hospital
NCT05858814	RC1012 Injection (Allo-DNT Cells) for the Prevention of Relapse in AML Patients After Allo-HSCT	AML	18–70 years	Guangdong Ruishun Biotech Co., Ltd.
NCT06707493	Ivosidenib as Post-HSCT Maintenance for AML	AML	18–75 years	Massachusetts General Hospital
NCT06754540	Azacitidine Combined with Donor Lymphocyte Infusion for Acute Myeloid Leukemia Post-transplant Relapse Prevention	AML	18–70 years	Shanghai General Hospital, Shanghai Jiao Tong University School of Medicine
NCT06765928	Selinexor Combined With Venetoclax Maintenance Therapy After Allo-HSCT	AML	18–75 years	Ruijin Hospital
NCT05768035	Safety and Efficacy of SMART101 in Adult Patients With Hematological Malignancies After Haploidentical HSCT With Post-transplant Cyclophosphamide	AML, ALL, MDS	≥18 years	Smart Immune SAS
NCT06783478	NK Cell Infusion for Remission Consolidation in AML: A Phase II Trial (NKLMA)	AML	18–75 years	Hospital de Clinicas de Porto Alegre
NCT06717958	Prospective Evaluation of Ivosidenib Maintenance Following Allogeneic Stem Cell Transplantation in Patients With Acute Myeloid Leukemia or High-risk Myelodysplastic Neoplasia With IDH1 (Isocitrate Dehydrogenase 1) Mutation (PIVOT)	AML, MDS	18–75 years	Technische Universität Dresden
NCT06704152	BSB-1001 in Patients Undergoing HLA-Matched Allogenic Hematopoietic Stem Cell Transplant for AML, ALL or MDS	AML, ALL, MDS	18–70 years	BlueSphere Bio, Inc.
NCT05528354	Venetoclax and Decitabine Based Conditioning Regimen Followed With Post-HSCT Decitabine Maintenance Therapy in TP53 Mutant AML/MDS Patients	AML, MDS	12–70 years	Zhejiang University
NCT06598384	VA as Maintenance Therapy Post Allo-HSCT in MDS and AML	AML, MDS	≥18 years	Navy General Hospital, Beijing
NCT03300492	Expanded Natural Killer Cells Following Haploidentical HSCT for AML/MDS	AML, MDS	≥18 years	University Hospital, Basel, Switzerland
NCT05796570	A Pilot Study to Evaluate the Feasibility of Post-Hematopoietic Stem Cell Transplant Prophylaxis with Decitabine Combined with Filgrastim for Children and Young Adults with AML, MDS and Related Myeloid Malignancies (MORE)	AML, MDS	1–39 years	Franziska Wachter
NCT03597321	Early Prophylactic Donor Lymphocyte Infusion After Allo-HSCT for Patients With AML (ELIT-AML01)	AML	18–70 years	Institut Paoli-Calmettes
NCT06441084	A Trial to Evaluate the Safety and Efficacy of NCR300 in Preventing Recurrence of Acute Myeloid Leukemia (AML) After Transplantation	AML	18–65 years	Nuwacell Biotechnologies Co., Ltd.
NCT02494167	Administration of Donor Multi TAA-Specific T Cells for AML or MDS (ADSPAM)	AML, MDS	child, adult	Baylor College of Medicine
NCT04849910	Allogeneic Engineered Hematopoietic Stem Cell Transplant (HCT) Lacking the CD33 Protein, and Post-HCT Treatment With Mylotarg, for Patients With CD33+ AML or MDS	AML, MDS	18–70 years	Vor Biopharma
NCT04128501	Venetoclax and Azacitidine for the Treatment of Acute Myeloid Leukemia in the Post-Transplant Setting	AML, ALL	18–75 years	M.D. Anderson Cancer Center
NCT06543381	Olutasidenib for the Treatment of Patients with IDH1 Mutated AML, MDS or CMML After Donor Hematopoietic Cell Transplant	AML, MDS, CML	≥18 years	City of Hope Medical Center
NCT05945849	CD33KO-HSPC Infusion Followed by CART-33 Infusion(s) for Refractory/Relapsed AML (CART33)	AML	≥18 years	University of Pennsylvania
NCT05233618	Study of Tagraxofusp for Post-Transplant Maintenance for Patients With CD 123+ AML, MF and CMML (HSCT 002)	AML, CML, MF	18–75 years	Karen Ballen, MD
NCT05682755	Chidamide Prevents Recurrence of High-risk AML After Allo-HSCT	AML	18–65 years	Sichuan University
NCT06158100	Venetoclax in Combination With Azacitidine (VEN/AZA) Followed by Donor Lymphocyte Infusion (DLI) for Patients With Very High-Risk Acute Myeloid Leukemia (AML) Undergoing Allogeneic Hematopoietic Cell Transplant (HCT)	AML	18–75 years	Antonio M Jimenez Jimenez
NCT06138587	Preemptive CIML NK Cell Therapy After Hematopoietic Stem Cell Transplantation	AML	≥18 years	Dana-Farber Cancer Institute
NCT06129734	Decitabine and Venetoclax Treatment as Maintenance Therapy in Patients Post Allograft Stem Cell Transplant	AML, MDS	≥18 years	Benjamin Tomlinson
NCT03533816	Expanded/Activated Gamma Delta T cell Infusion Following Hematopoietic Stem Cell Transplantation and Post-transplant Cyclophosphamide	AML, ALL, MDS, CML	19–65 years	University of Kansas Medical Center
NCT06498973	Tagraxofusp and Azacitidine for Maintenance Treatment in Patients With CD123 Positive AML and MDS Following Donor Hematopoietic Cell Transplant	AML, MDS	18–75 years	City of Hope Medical Center
NCT06575296	Revumenib for the Treatment of Acute Leukemia in Patients Post-Allogeneic Stem Cell Transplant	AML	≥2 years	City of Hope Medical Center
NCT03728335	Enasidenib as Maintenance Therapy in Treating Patients With Acute Myeloid Leukemia With IDH2 Mutation After Donor Stem Cell Transplant	AML	≥18 years	City of Hope Medical Center
NCT06532084	Sorafenib Relapse Prophylaxis After HCT With PTBCy Regimen (SoraGVL)	AML, MDS	18–75 years	St. Petersburg State Pavlov Medical University
NCT06440135	Ziftomenib Maintenance Post Allo-HCT	AML	≥18 years	Massachusetts General Hospital
NCT04764513	Safety and Efficiency of γδ T cell Against Hematological Malignancies After Allo-HSCT	AML, ALL	18–65 years	Chinese PLA General Hospital
NCT03843528	Vorinostat Dose-escalation After Allogeneic Hematopoietic Cell Transplantation	AML, MDS, JMML	1–21 years	Johns Hopkins All Children’s Hospital
NCT02782546	Cytokine Induced Memory-like NK Cell Adoptive Therapy After Haploidentical Donor Hematopoietic Cell Transplantation	AML	≥18 years	Washington University School of Medicine
NCT03856216	Inotuzumab Ozogamicin and Chemotherapy in Treating Patients With Leukemia or Lymphoma Undergoing Stem Cell Transplantation	ALL, lymphoma	12–70 years	M.D. Anderson Cancer Center
NCT06735690	Allogeneic CMV-Specific CD19-CAR T Cells Plus CMV-MVA Triplex Vaccine After Matched Related Donor Hematopoietic Cell Transplant for the Treatment of Patients With High-Risk Acute Lymphoblastic Leukemia	ALL	≥18 years	City of Hope Medical Center
NCT06438796	Blinatumomab Maintenance After Allo-HSCT	ALL	16–65 years	Ruijin Hospital
NCT05991973	Low-dose Chidamide Maintenance Therapy After Allo-HSCT for T cell Acute Lymphoblastic Leukemia or T cell Lymphomas	T-ALL/LL	14–70 years	Zhejiang University
NCT06686108	Demethylating Agents Combined with Venetoclax for High-risk T cell Lymphoma/Leukemia Post-Transplant Relapse Prevention	T-ALL/LL	14–55 years	Shanghai General Hospital, Shanghai Jiao Tong University School of Medicine
NCT06393985	Decitabine, Venetoclax and Blinatumomab for Maintenance Following HSCT in Patients With Ph-Negative B-ALL	ALL	18–65 years	The First Affiliated Hospital of Soochow University
NCT06658938	Blinatumomab as Maintenance Therapy in Patients With High-risk B-lineage Acute Lymphoblastic Leukemia Post Allogeneic Hematopoietic Cell Transplantation	ALL	≥14 years	Institute of Hematology & Blood Diseases Hospital, China
NCT06658925	Olverembatinib as Maintenance Therapy or Preemptive Therapy After Allo-HSCT in Ph+ALL	ALL	≥18 years	Institute of Hematology & Blood Diseases Hospital, China
NCT03622788	Cytokine-Treated Veto Cells in Treating Patients With Hematologic Malignancies Following Stem Cell Transplant	any heme malignancy	12–75 years	M.D. Anderson Cancer Center
NCT06075238	Blinatumomab Prevents Recurrence of R/R ALL After Allo-HSCT	ALL	16–65 years	Sichuan University
NCT04972942	Targeted Immunotherapy After Myeloablative TBI-Based Conditioning & AlloHCT in CAYA With High Risk T cell ALL & Lymphoma (ALLO-T-DART)	T-ALL	≤39 years	New York Medical College
NCT04746209	Blinatumomab After TCR Alpha Beta/CD19 Depleted HCT	ALL	≤25 years	Medical College of Wisconsin

In addition to the “what” of post-HSCT maintenance, questions still abound as to “who” ought to receive additional therapy and “how” this therapy should be administered (i.e., timing of initiation and duration of therapy) to appropriately balance the potential benefits of better disease control with toxicity concerns and impacts on immune reconstitution. Challenges will remain, including a lack of consensus regarding the duration of maintenance for many of the agents discussed as well as considering if and how different post-HSCT agents may affect decisions about the HSCT platform, such as conditioning regimen and graft selection. Additionally, as the field evolves to incorporate more targeted therapies in the upfront and relapsed settings, issues of antigen downregulation or escape will continue to necessitate a personalized approach. While much work is still to be done, recent advances in molecular and cellular-/immunotherapies provide hope that HSCT along with thoughtful maintenance therapy can one day be a means toward durable cure for all children and young adults with acute leukemias.

## Acknowledgments

The authors thank all of the investigators who have committed time and effort to performing the studies that comprise this review. The figures were generated using BioRender (https://biorender.com).

## Author contributions

A.W.R. developed the outline for the manuscript, performed the literature review, and wrote the first draft of the manuscript. A.K.K., R.F.A.-A., and M.S.T. reviewed and edited the manuscript. H.G.R. supervised the writing of the manuscript and reviewed the final version.

## Declaration of interests

The authors declare no competing interests.
